# Immunomodulatory effects of trastuzumab deruxtecan through the cGAS-STING pathway in gastric cancer cells

**DOI:** 10.1186/s12964-024-01893-3

**Published:** 2024-10-24

**Authors:** Kyoung-Seok Oh, Ah-Rong Nam, Ju-Hee Bang, Yoojin Jeong, Sea Young Choo, Hyo Jung Kim, Su In Lee, Jae-Min Kim, Jeesun Yoon, Tae-Yong Kim, Do-Youn Oh

**Affiliations:** 1 Cancer Research Institute, Seoul National University College of Medicine, 101 Daehak-Ro, Jongno-Gu, Seoul, 03080 Korea; 2 Integrated Major in Innovative Medical Science, Seoul National University Graduate School, Seoul, 03080 Korea; 3 Department of Internal Medicine, Seoul National University Hospital, Seoul, 03080 Korea

**Keywords:** ErbB-2, Trastuzumab deruxtecan, DNA damage, cGAS-STING signaling, Type-1 IFN

## Abstract

Although the efficacy of trastuzumab deruxtecan (T-DXd) against HER2-positive gastric cancers (GCs) has driven its clinical application, the precise mechanisms governing its immunomodulatory role remain unclear. In this study, we examined the immune-related mechanisms of action of T-DXd in GC cells. T-DXd exhibited potent antitumor effects in GC cells across diverse HER2 expression levels by inducing DNA damage and apoptosis. Activation of the DNA damage response by T-DXd led to increased PD-L1 expression. RNA-Seq analysis revealed that T-DXd modulated immune-related pathways, resulting in the upregulation of genes associated with inflammation and IFN signaling. Importantly, T-DXd activated the cGAS-STING pathway, inducing an IFN-I response in HER2-positive GC cells. Furthermore, T-DXd activated dendritic cells via the cancer cell-intrinsic cGAS-STING-IFN axis and enhanced PBMC-mediated tumor cell killing by activating CD8^+^ T cells. These findings provide valuable insights into the role of the cytosolic DNA sensing pathway in the action of T-DXd and offer a compelling rationale for combining T-DXd with immune checkpoint blockade therapies in GC treatment.

**Supplementary Information:**

The online version contains supplementary material available at 10.1186/s12964-024-01893-3.

## Background

HER2 is a key therapeutic target in gastric and gastroesophageal junction cancers, with approximately 20% of patients classified as HER2-positive (IHC3+ or IHC2+ and ISH+) [1]. Trastuzumab-deruxtecan (T-DXd), a HER2-targeted antibody–drug conjugate (ADC), consists of an anti-HER2 monoclonal antibody linked to a topoisomerase-I (TOP1) inhibitor payload via a cleavable tetrapeptide-based linker, with a drug-to-antibody ratio of approximately 8 [2, 3]. T-DXd has demonstrated superior antitumor efficacy and overall survival benefits in patients with gastric cancer (GC), particularly in those previously treated with trastuzumab [4]. Additionally, T-DXd also shows promising clinical efficacy in patients with HER2-low (IHC2+/ISH- or IHC1+) GC, addressing the unmet medical needs of patients with limited therapeutic options [5].

Clinical trials have demonstrated the efficacy of immune checkpoint inhibitors (ICIs), particularly in combination with chemotherapy, for GC [6–8]. Combining conventional targeted therapies like trastuzumab with ICIs has emerged as a promising strategy to enhance immune responses by reshaping the tumor microenvironment (TME) and expanding the patient population benefiting from immune checkpoint blockade (ICB) therapies [9].

Trastuzumab can enhance ICI efficacy through several immune-mediated mechanisms, such as activating natural killer cells, promoting intratumoral T cell infiltration, and upregulating PD-L1 and HLA-II expression in HER2-positive cancer cells [10–14]. ADCs, including T-DXd, may further augment ICI activity by inducing immunogenic cell death (ICD) and stimulating dendritic cell (DC) maturation [15–17]. The KEYNOTE-811 trial corroborated that incorporating pembrolizumab with trastuzumab and chemotherapy significantly improved the objective response rate of patients with HER2-positive GC, especially those with a PD-L1 combined positive score ≥ 1, underscoring the potential of this combination [18].

Preclinical studies have shown that combining T-DXd with ICIs enhanced antitumor immunity by inducing ICD and increasing the infiltration of DCs and CD8^+^ T cells into tumors [19, 20]. Furthermore, T-DXd has been reported to upregulate MHC-I, PD-L1, and chemokines involved in immune cell recruitment [19–21]. These findings support the clinical development of T-DXd in combination with ICIs, such as durvalumab, pembrolizumab, and PD-1/CTLA-4 bispecific antibody in the DESTINY-Gastric 03 trial (NCT04379596). However, the precise cellular mechanisms driving these immunomodulatory effects remain unclear, although DNA damage response (DDR) may play a role.

The cyclic GMP-AMP synthase (cGAS)-stimulator of interferon genes (STING) signaling pathway is a crucial, evolutionarily conserved mechanism in innate immunity that detects cytoplasmic double-stranded DNA (dsDNA) and triggers immune responses, primarily via type I interferon (IFN-I) signaling. This pathway is critical for recognizing and eliminating microbial pathogens and is increasingly recognized for its role in cancer biology, where it helps prevent immune evasion and promotes antitumor responses [22]. Cancer-intrinsic cGAS-STING activation can be initiated by several mechanisms, such as the loss of DNA repair factors or chromatin fragments resulting from chromosome instability, both of which can generate cytoplasmic dsDNA [23]. This activation is critical in preventing tumor immune escape by inducing an IFN-I response, which is known to stimulate immune surveillance mechanisms. In addition to its role in cancer cells, the cGAS-STING pathway can be activated in neighboring immune cells by dsDNA released from tumor cells, which primes DCs and subsequently activates CD8^+^ T cells [24]. Such activation of the cGAS-STING pathway in DCs via various cancer-derived factors is crucial for initiating adaptive immune responses and enhancing antitumor immunity, highlighting the potential of the cGAS-STING pathway in therapeutic strategies aimed at harnessing innate immune responses to fight cancers [25–27].

TOP1 inhibitors, which are employed as cytotoxic payloads in various ADCs, including T-DXd, have been shown to activate the cGAS-STING pathway. This activation may enhance antitumor immunity and potentially contribute to synergistic effects when combined with ICIs [28–33]. However, the exact pathways through which T-DXd influences immune regulation and inflammatory signaling remain to be fully elucidated.

Our study aims to deepen the understanding of the molecular mechanisms driving T-DXd-mediated immune modulation in GC cells, with a particular focus on how this therapy reshapes the TME to promote enhanced immune responses. By investigating these mechanisms, we seek to provide valuable insights that can guide future therapeutic strategies, particularly in combination with ICIs.

## Methods

### Human cell lines and reagents

The human leukemia monocytic cell line THP-1 (RRID:CVCL_0006), and 10 human GC cell lines; MKN45 (RRID:CVCL_0434), SNU-719 (RRID:CVCL_5086), SNU-601 (RRID:CVCL_0101), SNU-668 (RRID:CVCL_5081), SNU-638 (RRID:CVCL_0102), KATO-III (RRID:CVCL_0371), AGS (RRID:CVCL_0139), SNU-484 (RRID:CVCL_0100), NCI-N87 (RRID:CVCL_1603), and SNU-216 (RRID:CVCL_3946) were obtained from the Korean Cell Line Bank (Seoul, Korea) and cultured in RPMI 1640 medium (Welgene, Gyeongsan, Korea) supplemented with 10% FBS and 10 µg/mL gentamycin at 37 °C in 5% CO_2_. Trastuzumab and camptothecin were purchased from Roche Korea and CJ Healthcare (Daejeon, Korea), respectively. AZD6738 (an ATR inhibitor; AstraZeneca, UK) was purchased from Selleckchem (Korea), and AZD0156 (an ATM inhibitor; AstraZeneca) was kindly provided by AstraZeneca. T-DXd was obtained from Daiichi Sankyo (Tokyo, Japan). The ecto-nucleotide pyrophosphatase/phosphodiesterase (ENPP1) inhibitor (#29,809) was purchased from Cayman Chemical (Ann Arbor, MI, USA), and 2′3′-cGAMP (#SML1229) was obtained from Sigma-Aldrich (St. Louis, MO, USA). STING inhibitor H-151 (#inh-h151) was purchased from Invivogen (San Diego, CA, USA) and Human Type I IFN Neutralizing Antibody Mixture (#39,000) was purchased from PBL Assay Science (Piscataway, NJ, USA).

### Cell viability assay

Cells were seeded in 96-well plates and treated with 50 μl of MTT solution (Sigma-Aldrich). After incubation at 37 °C for 4 h, the liquid was discarded and MTT formazans were solubilized with DMSO. The absorbance was measured at 540 nm using a Multiskan GO spectrophotometer (Thermo Fisher Scientific, Waltham, MA, USA).

### Immunoblotting

Immunoblotting was performed according to an established protocol [34]. Briefly, cell extracts were prepared in SDS sample buffer, separated by polyacrylamide gel electrophoresis, and transferred onto a nitrocellulose membrane. The membrane was blocked with 1% nonfat milk and BSA in TBS-T for an hour. The primary antibodies used in this study were as follows: Santa Cruz Biotechnology (Dallas, TX, USA); anti-GAPDH (#sc-25778), anti-Chk2 (#sc-5278), Abcam (UK); anti-phospho ATM (S1981) (#ab81292), anti-ENPP1/PC1 (ab223268), anti-phospho Chk2 (T68) (#cst-2662), Millipore; anti-phospho histone H2A.X (γ-H2AX) (#05–636), Cell Signaling Technology (Danvers, MA, USA); anti-HER2/ErbB2 (#cst-2165), anti-ATM (#cst-2873), anti-cGAS (cst#83,623), anti-STING (cst#13,637), anti-phospho STING (S366) (#cst-19781), anti-phospho IRF3 (S396) (#cst4947), anti-phospho TBK1/NAK (S172) (#cst-5483), anti-phospho NF-kB p65 (S536) (#cst-3033), anti-phospho STAT1 (T701) (#cst-9167), anti-phospho STAT3 (T705) ( (#cst-9131), anti-IRF1 (#cst-8478), and anti-PD-L1 (#cst-13684).

### Subcellular fractionation

The isolation of cellular organelles from trypsinized cells was carried out using the Subcellular Protein Fractionation Kit (#78,840, Thermo Fisher Scientific) according to the manufacturer's guidelines.

### Clonogenic assay

Single-cell suspensions were seeded in six-well plates and incubated for nine days. Colonized cells were subsequently stained with Coomassie Brilliant Blue. Colony quantification was performed using the Gel Doc system software (Bio-Rad, Hercules, CA, USA).

### Cell cycle analysis

Cells were harvested by trypsinization and fixed in 70% ethanol at -20 °C for a minimum of 48 h. After treatment with RNase A, propidium iodide was added and the cell cycle was analyzed using a FACS Calibur flow cytometer (BD Biosciences, Franklin Lakes, NJ, USA).

### Comet assay

Single-cell gel electrophoresis (comet assay) was conducted as previously described [33]. Cells were embedded in agarose, lysed, and subjected to alkaline gel electrophoresis. DNA was stained with SYBR Green and visualized using a STELLARIS 5 confocal microscope.

### Immunofluorescence

Immunofluorescence was performed as previously described [34]. Cells were fixed with 4% paraformaldehyde and permeabilized with 0.5% Triton X-100. For detecting cytoplasmic dsDNA, a modified 0.01% Triton X-100 was used to selectively permeabilize the plasma membrane. Cells were incubated with primary antibodies, followed by incubation with secondary antibodies, and imaged using a STELLARIS 5 confocal microscope (Leica Microsystems). Detailed antibody information is as follows: Primary antibodies: anti-Topoisomerase I-DNA Covalent Complexes (TOP1cc) (Sigma-Aldrich, #MABE1084), anti-γ-H2AX (Millipore, #05–636), anti-cGAS (Santa Cruz Biotechnology, #sc-515777), anti-ENPP1 (Abcam, #ab223268), anti-Lamin B1 (Abcam, #ab16048), anti-dsDNA (Abcam, #ab27156), and anti-Rad51 (Santa Cruz Biotechnology, #sc-398587). Secondary antibodies: Alexa Fluor 488 goat anti-rabbit IgG (Invitrogen, #A-11008), Alexa Fluor 594 goat anti-rabbit IgG (Invitrogen, #A-11012), and Alexa Fluor 594 goat anti-mouse IgG (Invitrogen, #A-11032).

### In vivo Complex of Enzyme assay

Protein-DNA complexes were extracted using the Human Topoisomerase ICE Assay Kit (#TG1020-1, TopoGEN, Buena Vista, CO, USA) according to the manufacturer’s instructions. Cells were lysed, and genomic DNA was precipitated and washed before sonication. The DNA concentration was measured using a Nanodrop, and equal amounts were subjected to slot blot analysis. Protein-DNA complexes were filtered through nitrocellulose membranes, blocked with 5% dry milk in TBS-T, and incubated overnight with anti-Topoisomerase I-DNA Covalent Complexes antibody (#MABE1084, Sigma-Aldrich).

#### ELISA

Secretion levels of cGAMP, CCL5, and CXCL10 were measured using ELISA. Intracellular and extracellular cGAMP levels were assessed with the Direct 2’3’-Cyclic GAMP (cGAMP) ELISA Kit (Arbor Assays, #K067-H5). CCL5 and CXCL10 levels in conditioned media were quantified using the Human CCL5/RANTES Quantikine ELISA Kit (R&D Systems, #DRN00B) and Human CXCL10/IP-10 Quantikine ELISA Kit (R&D Systems, #DIP100), respectively. All assays were conducted following the manufacturer’s instructions.

### Exosome isolation and dsDNA quantification

Exosomes were isolated from conditioned media using Total Exosome Isolation Reagent (Thermo Fisher Scientific, #4,478,359) after culturing cells in RPMI 1640 medium containing 10% exosome-depleted FBS (Gibco, Grand Island, NY, USA, #A2720803). Exosomal DNA was isolated using the XCF Exosomal DNA Isolation Kit (System Biosciences, Palo Alto, CA, USA, #XCF200A-1) and quantified using the SpectraMax Quant AccuBlue Pico dsDNA Assay Kit (Molecular Devices, San Jose, CA, USA, #R8354) and agarose gel electrophoresis.

### RT-qPCR

Total RNA was extracted using TRIzol (Invitrogen, #10,296,028) and reverse-transcribed to cDNA with the ImProm-II™ Reverse Transcription System (Promega, #A3800). Real-time PCR was conducted on a QuantStudio 3 Real-Time PCR Instrument (Applied Biosystems) using TOPreal SYBR Green qPCR PreMIX (Enzynomics, #RT500M). Primer sequences for the target genes were as follows: IFNβ1: sense 5′-TTG ACA TCC CTG AGG AGA TTA AGC-3′, anti-sense 5′-TTA GCC AGG AGG TTC TCA ACA ATAG-3′; CXCL10: sense 5′-CCA TTC TGA TTT GCT GCC TTA TC-3′, anti-sense 5′-TAC TAA TGC TGA TGC AGG TAG AG-3′; IFIT1: sense 5′-GCC TAT CGC CAA GAT TTA GAT GA, anti-sense 5′-TTC TGG ATT TAA CCG GAC AGC-3′; ISG15: sense 5′-CGC AGA TCA CCC AGA AGA TCG-3′, anti-sense 5′-TTC GTC GCA TTT GTC CAC CA-3′; CCL5: sense 5′-CCA GCA GTC GTC TTT GTC AC-3′, anti-sense 5′-CTC TGG GTT GGC ACA CAC TT-3′; RIG1: sense 5′-ACC AGA CCT CCT CTT GGC-3′, anti-sense 5′-GAA GGG GCA GAT GGC TGT-3′; PD-L1 (CD274): sense 5′-CCA AGG CGC AGA TCA AAG AGA-3′, anti-sense 5′-AGG ACC CAG ACT AGC AGC A-3′; IL-12: sense 5′-AGT GTC AAA AGC AGC AGA GG-3′, anti-sense 5′-AAC GCA GAA TGT CAG GGA G-3′; actin: sense 5′-CCA ACC GCG AGA AGA TGA-3′, anti-sense 5′-CCA GAG GCG TAC AGG GAT AG-3′.

### RNA-Seq

Total RNA was isolated using TRIzol, and RNA integrity was assessed with TapeStation RNA ScreenTape (Agilent, #5067–5576). Only samples with a RIN > 7.0 were used for RNA library construction. Libraries were prepared with the TruSeq Stranded mRNA Sample Prep Kit (Illumina, #RS-122–2101), quantified using KAPA Library Quantification kits, and qualified via TapeStation D1000 ScreenTape (Agilent, #5067–5582) before sequencing on an Illumina NovaSeq platform. Raw reads were trimmed with Trimmomatic 0.38 and aligned to the GRCh38 reference genome using HISAT v2.1.0. StringTie v2.1.3b was employed for transcript assembly and read count calculations. Differential expression was analyzed using DESeq2 (|fold change|≥ 2, *p* < 0.05). Hierarchical clustering was conducted with complete linkage and Euclidean distance, and functional pathway analysis was performed using gProfiler.

### Gene set enrichment analysis 

Gene set enrichment analysis (GSEA) was performed using GSEA software version 4.3.2 (SCR_003199). Hallmark and Gene Ontology gene sets from version 7.4 of the molecular signature database (mSigDB) (RRID:SCR_016863) were used. The analysis was conducted with 1,000 permutations to calculate *p*-values.

### RNA interference

The cells were transfected with either cGAS-targeting or non-targeting control (scrambled) siRNAs (Genolution, Seoul, Korea) using Lipofectamine 2000 (Thermo Fisher Scientific) for 6 h. Twenty-four hours after the initial transfection, the cells were harvested and reseeded for further experiments. The siRNA sequences used in this study were: cGAS sense 5′-CUA AGA UGC UGU CAA AGU UUU-3′, anti-sense 5′-AAC UUU GAC AGC AUC UUA GUU-3′, IRF1 sense 5′-GCA UCA UGG UGG AUA UGG UUU-3′, and anti-sense 5′-AUU UCA CUG GCU UUG GAA CUU-3′.

### THP-1-derived DCs

THP-1 cells were differentiated into a functional DC-like phenotype by treatment with 20 ng/ml phorbol 12-myristate 13-acetate (PMA) (Bio-Techne, Minneapolis, MN, USA, #1201) and 20 ng/ml recombinant human IL-4 (PeproTech, #200–04) for 72 h. The medium was replaced with fresh cytokines on day 3. Cancer cells and THP-1-derived DCs were directly cocultured at a seeding ratio of 3:1. Antibodies against CD11c (#337,206), CD209 (#330,104), CD86 (#305,412), and HLA-DR (#307,610) were used to define the THP-1-derived DC population and evaluate their functional status, all of which were obtained from BioLegend (San Diego, CA, USA).

### PBMCs

PBMCs were isolated from peripheral blood using ficoll gradient centrifugation (Ficoll-Paque Plus, Cytiva, #17–1440-03) and cryopreserved until use. For functional analysis, thawed PBMCs were directly cocultured with cancer cells that were either pre-exposed to T-DXd or not. Brefeldin A (Biolegend, #420,601) was added 6 h prior to flow cytometry analysis, and intracellular staining of markers was performed using antibodies as follows: anti-CD3 (Invitrogen, #11–0037-42), anti-CD8a (Invitrogen, #17–0088-42), anti-CD340 (erbB2/HER-2) (Biolegend, #324,416), anti-Granzyme B (Biolegend, #372,218), anti-IFN gamma (Biolegend, #502,544), and anti-CD69 (Biolegend, #310,912). Zombie NIR™ Fixable Viability Kit (Biolegend, #423,105) was used to determine cell viability. A Cyto-Fast Fix/Perm Buffer Set (Biolegend, #426,803) was used for cell fixation and permeabilization. The gating strategy used for flow cytometry is shown in Figure S4.

### Analysis using the Tumor Immune Estimation Resource database

The Tumor Immune Estimation Resource (TIMER) 2.0 database, an online bioinformatics resource (http://timer.cistrome.org/) (RRID:SCR_018737), was employed to estimate the correlation between cGAS gene expression and immune infiltration levels within TCGA-STAD dataset [35].

### Statistical analysis

Statistical analyses were performed using GraphPad Prism (RRID:SCR_002798), version 8.0. Two-sided Student’s t-tests were used for all statistical analyses unless otherwise indicated in the figure legends. The threshold for statistical significance was set at *p* < 0.05.

## Results

### T-DXd induces DNA damage response and potent cytotoxic effects against GC cell lines with various HER2 expression levels

**Fig. 1:**
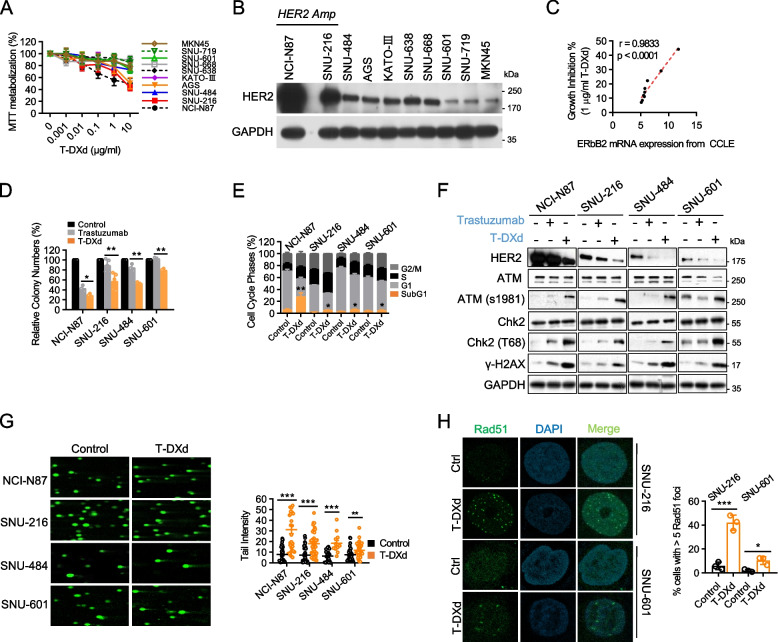
T-DXd induces DNA damage response and potent cytotoxic effects against GC cell lines with various HER2 expression levels. **A** GC cells were treated with varying concentrations of T-DXd for six days, and cell viability was measured using MTT assays. The results of four independent experiments are presented as the mean ± SEM of the percentage of viable cells. **B** Whole lysates were prepared from GC cells and subjected to immunoblotting to determine the basal expression level of HER2. **C** The nonparametric Spearman’s correlation between the growth inhibition rate of GC cells in response to T-DXd (Fig. 1A) and the mRNA expression level of HER2, obtained from the RNA-Seq-based CCLE expression dataset (DepMap Public 22Q2). **D** GC cells were treated with 1 µg/ml of trastuzumab or T-DXd for 10 days and subjected to clonogenic assays. Relative colony numbers are presented as mean ± SEM *, *p* < 0.05; **, *p* < 0.005 of three biological replicates. **E** The cell cycle distribution was evaluated by flow cytometry after five days of treatment with 1 µg/ml T-DXd in indicated GC cells, and data from at least three independent experiments are shown as mean ± SEM *, *p* < 0.05, **, *p* < 0.005. **F** GC cells treated with 1 µg/ml of trastuzumab or T-DXd for five days were prepared into whole lysates for immunoblotting. **G** Comet analysis was conducted in GC cells treated with 1 µg/ml of T-DXd for 72 h. Left panel: representative images of the three independent experiments are shown. Right panel: each scattered dot represents the tail intensity of single cells and the sum of three biological replicates. Data are shown as mean ± SEM **, *p* < 0.005, ***, *p* < 0.001. **H** Immunofluorescence analysis was performed to detect Rad51 foci. Left panel: GC cells treated with 1 µg/ml of T-DXd for 72 h were permeabilized with 0.5% Triton X-100 and stained for Rad51 (green), and counterstained with DAPI (blue). Representative images from three independent experiments are shown. Right panel: the relative number of cells positive for Rad51 foci was determined by counting over 100 cells in each dataset and shown as mean ± SD *, *p* < 0.05; ***, *p* < 0.001

We evaluated the antitumor effects of T-DXd using a panel of 10 GC cell lines. T-DXd elicited substantial inhibitory effects on the proliferation of GC cell lines with low HER2 expression (AGS and SNU-484), as well as those with high HER2 expression (NCI-N87 and SNU-216) (Fig. 1A and B). Importantly, we observed a strong correlation between the degree of growth inhibition induced by T-DXd and HER2 mRNA expression levels, indicating the HER2-dependent antiproliferative activity of T-DXd (Fig. 1C).

We selected four cell lines to represent distinct clinical HER2 status subtypes: NCI-N87 and SNU-216 (high HER2 with gene amplification) for HER2-positives, and SNU-484 (low HER2 with no copy number variation) and SNU-601 (extremely low HER2) for HER2-low and HER2-negative, respectively. T-DXd showed significant anti-growth effects even in HER2-low/negative cells, unlike trastuzumab. The mean percentage decrease in colony formation was: NCI-N87 (56.96% Tzb vs. 71.11% T-DXd); SNU-216 (10.94% Tzb vs. 42.74% T-DXd); SNU-484 (15.31% Tzb vs. 47.04% T-DXd); SNU-601 (-2.55% Tzb vs. 20.57% T-DXd) (Fig. 1D). Furthermore, T-DXd significantly augmented G2/M phase arrest and the SubG1 population in all four tested cell lines, indicating the induction of apoptotic cell death (Fig. 1E). These findings affirm the antitumoral action of T-DXd across GC cells with a broad spectrum of HER2 expression, including those traditionally considered unresponsive to anti-HER2 therapies.

To elucidate the mechanism underlying the effects of T-DXd, we investigated DNA damage pathways. T-DXd treatment induced the activation of the DDR pathway, as evidenced by the increased phosphorylation levels of ATM, Chk2, and histone H2A in all tested cell lines (Fig. 1F). Comet analysis further confirmed the presence of T-DXd-induced DNA double-strand breaks in all tested GC cell lines with various HER2 expression levels (Fig. 1G). Moreover, T-DXd treatment promoted the formation of Rad51 foci in the nucleus, indicating robust DNA damage induction (Fig. 1H).

### T-DXd-induced DDR activation regulates PD-L1 transcription through IRF1 in GC cells

**Fig. 2:**
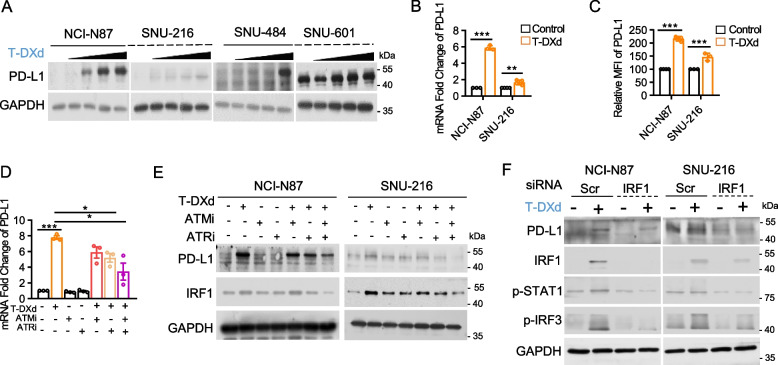
T-DXd-induced DDR activation regulates PD-L1 transcription through IRF1 in GC cells. **A** PD-L1 expression in GC cells treated with different concentrations of T-DXd (0, 0.04, 0.2, 1, and 5 µg/ml) for 120 h was assessed by immunoblotting. **B** Quantitative PCR analysis of PD-L1 expression in GC cells treated with 1 µg/ml of T-DXd for 72 h. β-Actin was used as a normalization control. Data are presented as mean ± SD ***, *p* < 0.001. **C** Flow cytometric analysis of surface PD-L1 expression in GC cells treated with 1 µg/ml of T-DXd for 72 h. Data from at least three independent biological replicates are shown as mean ± SD. ***, *p* < 0.001. **D** PD-L1 mRNA levels in NCI-N87 cells treated with T-DXd (1 µg/ml) and/or AZD0156 (ATMi, 0.1 µM), and/or AZD6738 (ATRi, 0.1 µM) for 72 h, were determined by RT-qPCR. Data from three independent experiments are represented as mean ± SEM *, *p* < 0.05; ***, *p* < 0.001. **E** Immunoblot analysis of PD-L1 and IRF1 expression levels in cell lysates from NCI-N87 cells treated with T-DXd (1 µg/ml) and/or inhibitors of ATM and ATR. **F** Immunoblot analysis of PD-L1 and IRF1-related gene expression levels in GC cells transfected with siRNAs targeting IRF1, with or without T-DXd treatment (1 µg/ml)

The DNA double-stranded break repair pathway, which is activated by various chemotherapeutic agents, plays a specific role in the regulation of PD-L1 expression in cancer cells [36]. Therefore, we examined the effect of the T-DXd-induced activation of the DDR pathway on PD-L1 expression in GC cells. T-DXd treatment notably increased PD-L1 expression in GC cells, regardless of their HER2 status, even in those with low HER2 expression (Fig. 2A). Using HER2-amplified GC cell lines, we demonstrated that T-DXd treatment upregulated PD-L1 at the transcriptional level and enhanced the surface retention of PD-L1 (Fig. 2B and C). To explore the involvement of the DDR pathway in T-DXd-induced PD-L1 regulation, we used ataxia-telangiectasia mutated (ATM), Ataxia-telangiectasia and Rad3-related (ATR) inhibitors, which are both critical regulators of the DDR pathway. Our findings revealed that ATR inhibition partially counteracted the T-DXd-induced transcriptional upregulation of PD-L1, whereas dual inhibition of ATM and ATR more effectively abolished T-DXd-mediated PD-L1 upregulation (Fig. 2D). Consistent with these observations, immunoblotting assays confirmed that the dual inhibition of ATM and ATR prevented T-DXd from enhancing PD-L1 expression in GC cells (Fig. 2E). Remarkably, the level of IRF1, a transcription factor implicated in Chk1-mediated PD-L1 transcriptional regulation, correlated with PD-L1 expression (Fig. 2E). Using siRNA-mediated knockdown of IRF1, we further validated that IRF1 deficiency attenuated the T-DXd-induced increase in PD-L1 expression (Fig. 2F). Collectively, our findings suggest that T-DXd-induced activation of the DDR pathway regulates PD-L1 transcription through IRF1.

### T-DXd provokes a gene expression signature related to enhanced tumor immunogenicity in GC cell lines with high and low HER2 expression

**Fig. 3:**
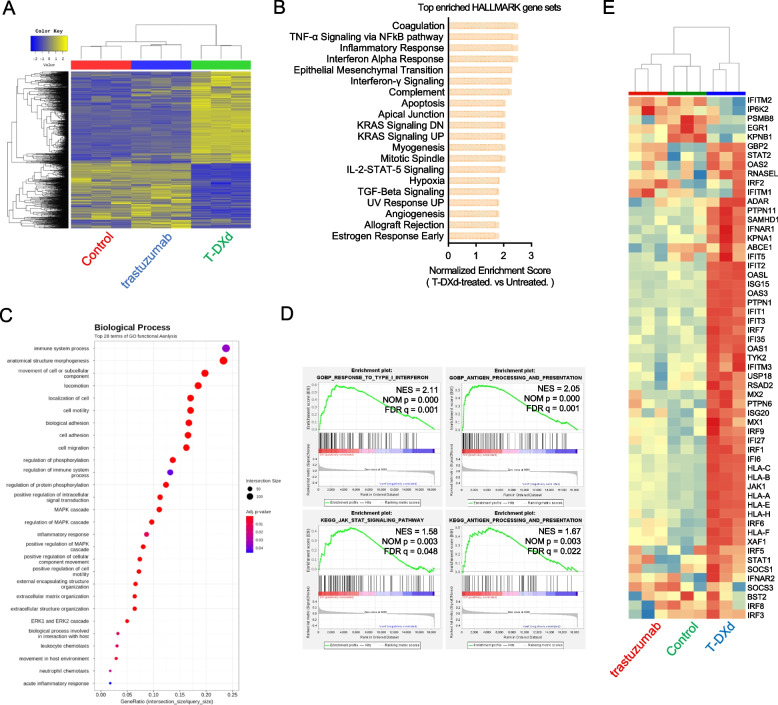
T-DXd provokes a gene expression signature related to enhanced tumor immunogenicity in GC cell lines with high and low HER2 expression. RNA-Seq was performed using NCI-N87 cells after treatment with the vehicle, trastuzumab (1 µg/ml), and T-DXd (1 µg/ml) for 72 h. **A** Hierarchically clustered heatmap of 1,310 DEGs (| fold change |≥ 2 and raw *p* < 0.05, *n* = 3). **B** The top 20 enriched Hallmark gene sets in response to T-DXd ranked by normalized enrichment score from GSEA. **C** The result of the GO enrichment analysis showing the top 28 enriched gene sets following T-DXd treatment. **D** Representative images of GSEA showing the enrichment of gene signatures associated with antitumor immune response by T-DXd treatment. **E** Heatmap illustrating Reactome Interferon alpha/beta signaling geneset from RNA-Seq data

To further investigate the mechanism of action of T-DXd in GC, we conducted transcriptome analysis using RNA-Seq in high-HER2-expressing NCI-N87 and low-HER2-expressing SNU-484 cells. In the NCI-N87 dataset, we identified 1,010 differentially expressed genes (DEGs) with 675 genes upregulated and 335 genes downregulated by T-DXd treatment, using a threshold of |FC|≥ 2 and a raw *p*-value < 0.05 (Fig. 3A). T-DXd induced a transcriptional landscape distinct from that induced by trastuzumab, underpinning the specific effects of TOP1 inhibition (Fig. 3A). Seven of the top 20 ranked, enriched hallmark gene sets upregulated by T-DXd treatment were involved in antitumor immunity, including *Inflammatory Response* and *Interferon Alpha Response* (Fig. 3B). GO functional analysis and GSEA revealed the enrichment of biological processes genesets associated with antitumor immune modulation, such as *response to type I interferon* (GO:0034340) and *antigen processing and presentation* (GO:0030333) following T-DXd treatment (Fig. 3C and D). Notably, the induction of gene expression linked to IFN-I signaling was exclusively achieved by T-DXd, not trastuzumab (Fig. 3E).

In SNU-484 cells, T-DXd showed potent immunomodulatory effects, as evidenced by the highest normalized enrichment score of the hallmark gene sets *interferon alpha response* and *interferon-gamma signaling* (Fig. S1A). Gene sets associated with *response to type I interferon* (GO:0034340) and *antigen processing and presentation* (GO:0030333) were also significantly enriched by T-DXd treatment in SNU-484 cells, suggesting that the immunomodulatory effects of T-DXd are not exclusively limited to HER2-positive GC (Fig. S1B and C).

Collectively, our findings suggest that T-DXd modulates gene expression linked to tumor immunogenicity in GC cells, potentially driving its antitumor effects by reprogramming the TME of GC.

### T-DXd activates the cytosolic DNA recognition pathway in GC cells

**Fig. 4:**
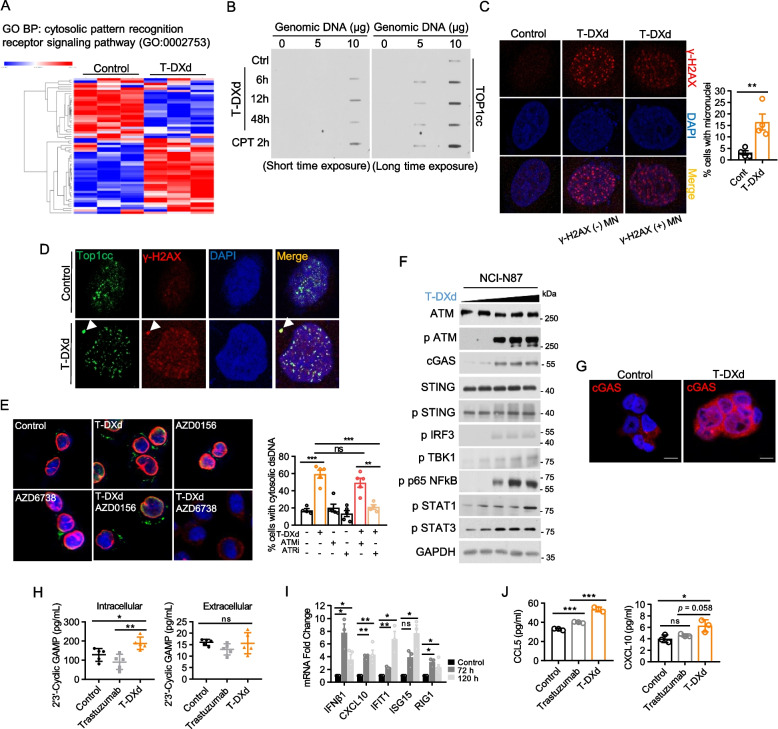
T-DXd activates the cytosolic DNA recognition pathway in GC cells. **A** Heatmap of cytosolic pattern recognition receptor pathway genes showing differential expression after T-DXd treatment. **B** NCI-N87 cells treated with 1 µg/ml of T-DXd or 10 µM of camptothecin for indicated times were subjected to slot blot assays to measure TOP1cc levels. Representative images from biological duplicates are shown. **C** Immunofluorescence analysis was conducted in SNU-216 cells treated with 1 µg/ml of T-DXd for 72 h to detect the formation of DNA damage foci and MN. Permeabilized cells were stained for γ-H2AX (red) and DNA compartment (blue). Left panel: representative images from four independent experiments are shown, indicating the γ-H2AX-positive MN and γ-H2AX-negative MN. Right panel: the percentage of cells with MN was measured by counting more than 100 cells of each experiment and shown as mean ± SEM **, *p* < 0.005. **D** Immunofluoresence analysis using SNU-216 cells treated with 1 µg/ml of T-DXd for identifying the TOP1cc (green) formation and its colocalization with MN. **E** Immunofluorescence analysis of cytoplasmic dsDNA (green) in SNU-216 cells treated with 1 µg/ml of T-DXd, 0.1 µM AZD0156 (ATR inhibitor), 0.1 µM AZD6738 (ATM inhibitor), or their combination with T-DXd for 48 h. Nuclei were counterstained with DAPI (blue), and Lamin B1 (red) was used as a nuclear envelope marker. Left panel: representative images from four biological replicates are shown. Right panel: the percentage of cells positive for cytoplasmic dsDNA is shown as mean ± SEM. ns: not significant; **, *p* < 0.005; ***, *p* < 0.001. **F** Immunoblotting of genes associated with the canonical cGAS-STING pathway in NCI-N87 cells treated with varying concentrations of T-DXd (0, 0.04, 0.2, 1, and 5 µg/ml). **G** Immunofluorescence analysis of SNU-216 cells treated with 1 µg/ml of T-DXd showing the upregulation of cGAS (Red) expression. The nuclei were counterstained by DAPI (blue) and representative images from three biological replicates are shown. **H** The synthesis (left panel) and secretion (right panel) levels of cGAMP were determined by ELISA in NCI-N87 cells treated with 1 µg/ml of trastuzumab or T-DXd for 72 h. Every symbol represents one biological replicate from five experiments. Statistical significance is shown as *, *p* < 0.05; **, *p* < 0.005. **I** Quantitative PCR analysis determined the gene expression level of ISGs in NCI-N87 cells in response to 1 µg/ml of T-DXd treatment. Each dot represents one biological replicate and data are shown as mean ± SEM, ns: not significant, *, *p* < 0.05; **, *p* < 0.005. **J** Secretion levels of CCL5 (left panel) and CXCL10 (right panel) in NCI-N87 cells treated with 1 µg/ml trastuzumab or T-DXd, measured by ELISA

To elucidate the mechanism underlying T-DXd-induced IFN-I response in GC cells, we investigated the involvement of the cytosolic DNA recognition pathway, specifically focusing on cGAS, which has been shown to interact with TOP1cc and trigger a proinflammatory response [37]. Using RNA-Seq data from NCI-N87 cells, we found that T-DXd effectively altered the gene expression profile associated with the cytosolic DNA recognition signaling pathway (Fig. 4A). T-DXd treatment led to the formation of TOP1cc, peaking at 12 h post-treatment, and the generation of micronuclei (MN), small cytoplasmic fragments derived from nuclear chromosomes (Fig. 4B and C). About 10% of these MN colocalized with extranuclear γ-H2AX foci, and both γ-H2AX-negative and γ-H2AX-positive MN colocalized with TOP1cc, suggesting that T-DXd-induced TOP1cc-mediated DNA damage contributes to MN formation (Fig. 4D). Aligned with this, T-DXd promoted the formation of cytosolic dsDNA, which was inhibited by ATR but not by ATM inhibiton (Fig. 4E). This suggests that single-strand breaks (SSBs) caused by trapped TOP1cc by T-DXd are detected by ATR, leading to the release of genomic DNA into the cytoplasm. This observation is consistent with the finding that γ-H2AX-positive MN represented only about 10% of the total MN, emphasizing the role of ATR in regulating the cytoplasmic leakage of dsDNA after T-DXd treatment.

We found T-DXd upregulated cytoplasmic cGAS expression and activated canonical cytosolic DNA recognition signaling cascades, as evidenced by increased phosphorylation of STING, IRF3, TBK1, NFkB, STAT1, and STAT3 (Fig. 4F and G). Furthermore, ELISA confirmed that T-DXd increased the intracellular production of 2′3′-cyclic GMP-AMP (cGAMP), an immunotransmitter that is produced by cGAS and activates the adaptor protein STING (Fig. 4H). The activation of IFN-I response promoted by T-DXd was demonstrated by increased mRNA transcription of central ISGs (IFNβ1, CXCL10, IFIT1, ISG15, and RIG1) and increased secretion of chemokines such as CCL5 and CXCL10, which are associated with the recruitment of CD8^+^ T cells and effective antitumor immunity (Fig. 4I and J).

### cGAS is essential for the T-DXd-mediated promotion of IFN-I response in GC cells

**Fig. 5:**
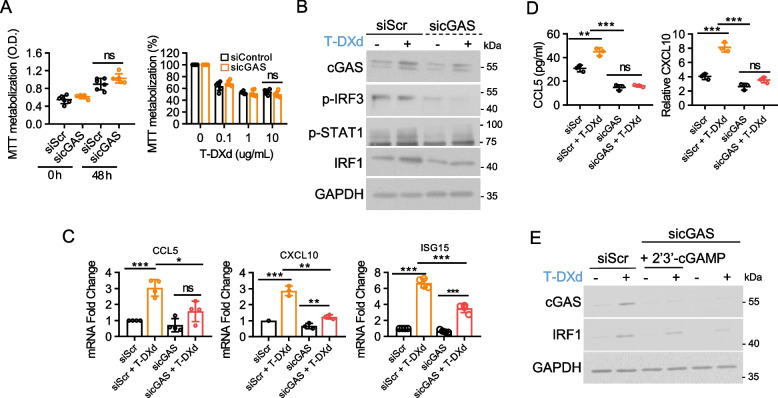
cGAS is essential for the T-DXd-mediated promotion of IFN-I response in GC cells. **A** The impact of cGAS knockdown on cell proliferation (left panel) and sensitivity to T-DXd (right panel) was assessed by MTT assays using NCI-N87 cells transfected with the indicated siRNAs. Data represent the mean ± SD from three independent experiments, ns: not significant. **B** The immunoblot assay determined the effect of cGAS deficiency on T-DXd-induced STAT1 pathway activation in NCI-N87 cells. **C** Quantitative PCR analysis evaluated the mRNA expression levels of ISGs in NCI-N87 cells transfected with specific siRNAs. Data represent the mean ± SD from three independent experiments, ns: not significant, *, *p* < 0.05; **, *p* < 0.005; ***, *p* < 0.001. **D** ELISA measured the cytokine release from cancer cells transfected with specific siRNAs in response to T-DXd treatment. The relative secretion levels of CCL5 and CXCL10 are depicted as mean ± SD, ns: not significant, **, *p* < 0.005; ***, *p* < 0.001. **E** Immunoblotting was employed to evaluate the restoration of T-DXd-induced IRF1 induction, which was attenuated by cGAS depletion upon cGAMP transfection in NCI-N87 cells

Considering the crosstalk between multiple pathways in the regulation of IFN-I, we investigated whether the cGAS-STING pathway plays a specific role in the T-DXd-induced IFN-I response. cGAS knockdown suppressed T-DXd-induced phosphorylation of IRF3 and STAT1, as well as the upregulation of IRF1, a downstream target gene of STAT1 signaling, while having a minimal effect on cell proliferation (Fig. 5A and B). Furthermore, cGAS depletion prevented the transcriptional upregulation of ISGs and abolished the T-DXd-induced enhanced secretion of CCL5 and CXCL10 (Fig. 5C and D). Moreover, transfection with cGAMP partially rescued T-DXd-induced IRF1 expression, which was suppressed by cGAS depletion (Fig. 5E). These observations demonstrate the crucial role of the cGAS-STING pathway in the activation of the proinflammatory IFN-I response by T-DXd.

### T-DXd induces DC activation and enhances PBMC-mediated tumor cell killing

**Fig. 6:**
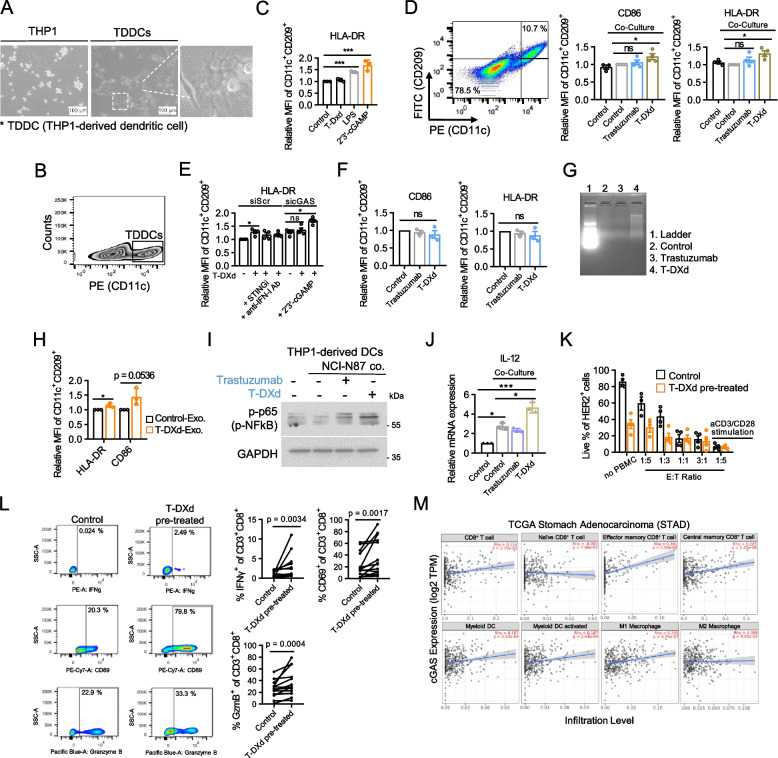
T-DXd induces DC activation and enhances PBMC-mediated tumor cell killing. **A** THP-1 cells were differentiated into TDDCs by adding a cocktail of 20 ng/ml of PMA and recombinant human IL-4. The morphologies of THP-1 cells and TDDCs were observed under a light microscope. **B** Flow cytometry analysis was performed to determine the relative proportion of CD11c^+^ cells in TDDCs at day 6 post-differentiation. **C** Flow cytometry analysis was performed to assess the expression level of HLA-DR in CD11c^+^/CD209^+^ TDDCs treated with the vehicle, T-DXd, LPS, or transfected with 2 µg/ml of cGAMP. **D** NCI-N87 cells and TDDCs were cocultured at a ratio of 3:1 for 72 h with 1 µg/ml of trastuzumab or T-DXd. Flow cytometry analysis was performed to determine the expression level of HLA-DR in CD11c^+^/CD209^+^ TDDCs. **E** NCI-N87 cells transfected with specific siRNAs were pretreated with T-DXd in the presence of STINGi (H-151, 1 µg/ml), human type I IFN neutralizing antibody mixture, or cGAMP transfection for 6 h. Subsequently, cells were harvested and cocultured with TDDCs for an additional 72 h. Flow cytometry analysis was performed to determine the expression level of HLA-DR in CD11c^+^/CD209^+^ TDDCs. **F** TDDCs were generated from THP-1 cells in the lower chambers of Transwell plates. NCI-N87 cells were seeded in the upper chambers of Transwell plates and treated with trastuzumab or T-DXd (1 µg/ml) for 72 h. Flow cytometry analysis determined the expression level of CD86 (left panel) and HLA-DR (right panel) in CD11c^+^/CD209^+^ TDDCs. Data from three independent experiments are presented as mean ± SEM, ns: not significant. **G** Agarose gel electrophoresis of exosomal dsDNA isolated from the culture medium of NCI-N87 cells treated with 1 µg/ml of T-DXd. Exosomes were isolated from the conditioned medium, followed by dsDNA enrichment. Representative images from two independent experiments are shown. **H** Bar graph showing the expression levels of CD86 and HLA-DR in CD11c^+^/CD209^+^ TDDCs after 48 h incubation with exosomes isolated from the culture medium of NCI-N87 cells treated with T-DXd. Flow cytometry was performed to assess the activation markers. Data represent the mean ± SEM from three independent experiments. *, *p* < 0.05. **I** An immunoblot assay was performed on lysates from TDDCs after 48 h of indirect coculture with NCI-N87 cells in the presence of 1 µg/ml of trastuzumab or T-DXd. **J** RT-qPCR was used to measure the expression levels of IL-12 in TDDCs that were indirectly cocultured with NCI-N87 cells for 48 h in the presence of 1 µg/ml of trastuzumab or T-DXd. Data from three independent experiments were shown as mean ± SD, *, *p* < 0.05; ***, *p* < 0.001. **K** PBMC-mediated killing assay of NCI-N87 cells pretreated with T-DXd or the vehicle. Following a 72-h coculture, flow cytometry was used to determine the percentage of live HER2^+^ cells. **L** Flow cytometry analysis of CD69 expression, as well as intracellular staining of Granzyme B and IFN-γ in CD8^+^ T cells from PBMCs and NCI-N87 cocultures, with or without T-DXd pre-treatment. Left panel; representative images from at least 11 experiments. Right panel; frequencies of CD8^+^ T cells expressing CD69, Granzyme B, and IFN-γ. Statistical significance was determined using a paired Wilcoxon test. **M** Scatterplots illustrating the correlation between cGAS expression (log2TPM) and the tumor infiltration of immune cells in TCGA-STAD, including subsets of CD8^+^ T cells, DCs, and macrophages, analyzed using the TIMER2.0 tumor immune estimation resource

Emerging evidence suggests that cancer cell-derived DNA can enter the cytosol of DCs and enhance the cGAS-STING-dependent IFN-I response, leading to DC maturation and potent antitumor immune responses [24]. Additionally, cGAMP, produced upon cGAS-STING activation, can be transferred to neighboring DCs, further activating the STING pathway in these bystander cells and amplifying antitumor immune responses. [25, 27]. To explore the effects of T-DXd on DCs, we employed a reliable in vitro model using THP-1 cells. We differentiated THP-1 cells into THP-1-derived DCs (TDDCs) through PMA and IL-4 stimulation and successfully recapitulated the essential features of DCs, as corroborated by the expression of a DC-specific marker (Fig. 6A and B) [38]. Additionally, we validated the functionality of TDDCs by observing the upregulation of the DC activation marker HLA-DR upon cGAMP transfection or LPS treatment (Fig. 6C). These findings confirm the physiological response of the TDDC model and establish its suitability for further immunological investigations. T-DXd, but not trastuzumab, enhanced CD86 and HLA-DR expression in TDDCs in the coculture setting of NCI-N87 cells and TDDCs, indicating an indirect immunomodulatory effect of T-DXd on DC functionality through cancer cells (Fig. 6D). To elucidate the role of the cancer cell-intrinsic cGAS-STING pathway in the upregulation of DC activation markers induced by T-DXd, we performed coculture experiments using NCI-N87 cells pretreated with a STING inhibitor or an IFN-I neutralizing antibody. Remarkably, pharmaceutical inhibition of cancer cell-intrinsic STING or IFN-I significantly mitigated the T-DXd-induced augmentation of HLA-DR expression in TDDCs (Fig. 6E). Moreover, cGAS knockdown in NCI-N87 cells yielded similar results, further underscoring the involvement of the cGAS-STING pathway in T-DXd-mediated DC activation (Fig. 6E). cGAMP transfection in NCI-N87 cells rescued the upregulation of HLA-DR in TDDCs following T-DXd treatment, which was attenuated in the absence of cancer cell-intrinsic cGAS (Fig. 6E). This finding suggests that cGAMP, an activator of the cGAS-STING pathway, can restore DC activation, which is compromised by the loss of intrinsic cGAS in cancer cells. In the transwell system, T-DXd failed to induce cGAS-STING pathway activation or upregulation of CD86 and HLA-DR in TDDCs where direct physical interaction between cancer cells and DCs was prevented (Fig. 6F, S2A-C). This suggests that soluble factors, including released dsDNA, were insufficient to activate DCs. Given that exosomal DNA is too large to pass through the transwell pores, we shifted our focus to exosomes. Evidence indicates that exosomes can transfer nuclear DNA and other immunostimulatory molecules to immune cells, potentially driving DC activation [32, 39, 40]. Indeed, T-DXd treatment potentiated the release of exosomal DNA from cancer cells, and these exosomes effectively activated DCs, leading to the upregulation of HLA-DR and a trend toward increased CD86 expression, although the latter did not reach statistical significance (*p* = 0.0536) (Fig. 6G, S3, H). This suggests that physical interaction or vesicle-mediated transfer of nuclear material, such as exosomal DNA, may be crucial for DC activation. Additionally, we found that secretory factors from cancer cells in response to T-DXd were sufficient to augment NFkB phosphorylation and IL-12 production, suggestive of DC maturation and potential induction of Th1 response (Fig. 6I and J). Collectively, these data suggest that T-DXd may modulate DC function in a cancer cell-dependent manner, particularly through the cGAS-STING-IFN-I axis.

To further investigate the immunomodulatory effects of T-DXd, we optimized an in vitro 2D coculture system using NCI-N87 cells and human PBMCs obtained from healthy donors. NCI-N87 cells treated with either T-DXd or the vehicle were cocultured with PBMCs at varying effector cell (E) to target cell (T) ratios. We observed a direct correlation between the number of effector cells and PBMC-mediated tumor cell eradication (Fig. 6K). To optimize subsequent coculture investigations, we used a 1:3 ratio of immune cells to target cells, which notably augmented T-DXd-induced tumor cell death (Fig. 6K). To unveil the precise contribution of T-DXd to immune-mediated tumor cell killing, we assessed the functional profile of CD8^+^ T cells. Remarkably, we observed an increased proportion of CD8^+^ T cells expressing activation markers CD69, Granzyme B, and IFN-γ exclusively in the presence of T-DXd-treated cancer cells (Fig. 6L). These results underscore the capacity of T-DXd to induce ICD within cancer cells while concurrently invigorating CD8^+^ T cells, ultimately resulting in the orchestrated eradication of tumor cells.

Finally, using the TIMER2.0 immune estimation database, we established an association between cGAS expression and immune cell infiltration in patients with GC. cGAS expression positively correlated with the infiltration of myeloid DCs, specifically with its activated subset, as well as M1 macrophages (Fig. 6M). Both populations are intricately linked to Th1 response and robust antitumor immunity. Additionally, tumor-infiltrating effector memory and central memory CD8^+^ T cells, but not naïve CD8^+^ T cells (which play a bystander role in tumor killing), correlated with cGAS levels (Fig. 6M). These observations substantiate the clinical relevance of our in vitro results and underscore the pivotal role of cGAS in shaping the action of T-DXd, effectively connecting tumor-intrinsic immunogenicity with the adaptive immune response against GCs.

## Discussion

In this study, we uncovered key mechanisms underlying the antitumor effects of T-DXd in GC cells, particularly its role in modulating immune checkpoint signaling and antitumor immunity. We found that T-DXd induces HER2-dependent antiproliferative effects by causing DNA damage and apoptosis in HER2-expressing GC cells, which led to the upregulation of PD-L1 via IRF1. Transcriptomic analysis revealed that T-DXd enhances the expression of IFN-I signature genes in HER2-expressing GC cells. T-DXd promoted cGAS-STING pathway activation through TOP1cc, leading to IFN-I production, DC activation, and enhanced CD8^+^ T cell-mediated tumor killing.

Notably, T-DXd upregulated PD-L1 expression in HER2-low as well as HER2-positive GC cells, expanding its clinical significance. Given the role of PD-L1 as a key biomarker for ICI response, this finding highlights new therapeutic opportunities for patients with HER2-low GC, where 40–60% of HER2-negative cases lack effective targeted treatments. The concurrent induction of IFN-I signature genes further supports the rationale for combining T-DXd with ICIs to enhance antitumor immunity in this patient population.

TOP1 inhibitors like T-DXd induce DNA damage primarily by creating SSBs via the accumulation of TOP1cc. If unrepaired, these SSBs can lead to DSBs during replication [41]. ATR, activated by SSBs, is crucial for managing TOP1cc-induced damage, and it is generally assumed that SSBs drive cytosolic dsDNA accumulation, leading to immune activation via the cGAS-STING pathway. However, a recent study has shown that TOP1 poisons can also cause R loop-mediated genome instability, where ATM, rather than ATR, is more critical [42]. This raises the possibility that both ATR and ATM might contribute to cytosolic dsDNA formation depending on the type of DNA damage. Our results indicate that T-DXd-induced cytosolic dsDNA formation is predominantly mediated by ATR. The limited presence of γ-H2AX-positive MN in our experiments suggests that cytosolic dsDNA is primarily linked to SSBs rather than DSBs, further supporting the role of ATR in this process. The minimal involvement of ATM implies that R-loop formation may not play a major role in the mechanism of action of T-DXd. However, the precise mechanisms governing the leakage of nuclear DNA into the cytoplasm and the putative interplay between MN and cytosolic dsDNA remain unclear.

DNA-damaging agents, including T-DXd, can activate both canonical and non-canonical STING pathways. The non-canonical STING pathway involves ATM-interferon gamma-inducible protein 16 (IFI16)-mediated DDR pathway, which operates independently of cGAS [43]. In our study, ATM inhibition partially reduced T-DXd-induced cGAS-STING pathway activation and ISG upregulation, suggesting involvement of both pathways (Fig. S5A and B). While ATR played a critical role in cytosolic dsDNA formation, ATM specifically senses nuclear DSBs, indicating a dual role for cytosolic and nuclear DNA damage in T-DXd-induced STING activation.

Our study identified a significant increase in exosomal DNA released from T-DXd-treated GC cells, which can possibly be explained by the packaging of nuclear content from MN into exosomes during genomic instability [44]. However, the presence of a specific subpopulation of exosomes carrying nuclear DNA has yet to be confirmed, and it remains unknown whether the cGAS pathway directly contributes to exosome biogenesis or regulation. Exosomes released by T-DXd-treated cells induced HLA-DR and CD86 upregulation in TDDCs, suggesting a role for exosomal DNA in immune activation. Interestingly, direct coculture experiments revealed that cGAS-STING activation in cancer cells was necessary for TDDC activation, raising questions about the interplay between exosomal DNA and the cGAS-STING pathway. Future studies are required to clarify whether exosomes from cGAS-depleted cells retain their capacity to activate DCs and whether distinct exosome subpopulations contribute disproportionately to immune activation.

We also observed that T-DXd-induced HLA-DR expression in DCs was inhibited by anti-IFN-I antibodies, indicating that the cGAS-STING pathway in cancer cells indirectly drives DC activation through IFN-I. However, when transwell assays were performed, DC activation was abolished, suggesting that physical interactions or the direct transfer of molecules, such as cGAMP, between cancer cells and DCs may be required for full immune activation. The complex interplay between cGAS-STING activation and soluble factors in the TME requires further investigation to clarify the precise mechanisms underlying these interactions.

Recent studies have identified key regulatory factors within the cGAS-STING pathway that could serve as potential therapeutic targets [45]. One such factor is ENPP1, a transmembrane hydrolase that degrades extracellular cGAMP and produces immunosuppressive adenosine [46–48]. Our analysis revealed that T-DXd treatment significantly elevated ENPP1 levels, particularly in the cell membrane, indicating its potential role as a negative regulator of T-DXd-induced cGAMP signaling (Fig. S6A-C). Investigating the co-targeting of ENPP1 alongside T-DXd could be a promising approach to enhance immunostimulatory effects by preventing cGAMP degradation and amplifying cGAS-STING pathway activation.

HER2 has been implicated in suppressing innate immunity by blocking STING-TBK1 interactions, thus impairing cGAS-STING pathway activation [49]. Our data suggest that T-DXd may enhance the cGAS-STING pathway through dual mechanisms: freeing STING from HER2 inhibition and increasing cytosolic DNA via TOP1cc. While the antitumor function of the cGAS-STING-mediated IFN-I response is clear, this pathway also has dichotomous effects on the TME. Previous studies have shown that cGAS-STING can promote metastasis and tumor progression in certain contexts, likely through chronic inflammation and recruitment of myeloid-derived suppressor cells [50, 51]. Additionally, chronic inflammation during senescence has been shown to promote tumor progression through the recruitment of myeloid-derived suppressor cells [52]. Moreover, cGAS exhibits STING-independent functions in the nucleus, such as regulating DNA repair and cellular senescence [53]. These observations highlight the need for a nuanced approach in targeting the cGAS-STING pathway, considering both its antitumor potential and its context-dependent protumorigenic effects.

Finally, while our TDDC model effectively recapitulates key features of DCs, and PBMCs were employed to assess immune activation, the broader, systemic role of STING pathway in the intact TME remains unexplored. Future studies using humanized mouse models or syngeneic tumor models expressing human HER2 will be necessary to fully evaluate these interactions.

## Conclusions

Our study provides comprehensive insights into the mechanisms underlying the antitumor effects of T-DXd in GC. T-DXd induces the DDR, activates an immune response through the cGAS-STING pathway, and enhances DC activation and PBMC-mediated tumor cell killing. These findings highlight the multifaceted immunomodulatory properties of T-DXd and support its potential as an effective therapeutic strategy in combination with ICB therapies in patients with HER2-positive GC.

## Supplementary Information

Supplementary Material 1.

## Data Availability

The RNA-Seq data produced in this study is accessible via NCBI GEO under the accession number GSE245851.

## Abbreviations

ADC: Antibody-Drug Conjugate
DDR: DNA Damage Response
DSB: Double-Strand Breaks
TOP1: DNA topoisomerase 1
TOP1cc: Topoisomerase-1 DNA covalent cleavage complex
ATM: Ataxia-telangiectasia mutated
ATR: Ataxia telangiectasia and Rad3-related protein
Rad51: DNA repair protein RAD51 homolog 1
Chk1: Checkpoint kinase 1
Chk2: Checkpoint kinase 2
cGAS: Cyclic GMP-AMP Synthase
STING: Stimulator of interferon genes
IFN-I: Type-I Interferons
MN: Micronuclei
DEG: Differentially Expressed Gene
GSEA: Gene Set Enrichment Analysis
IRF1: Interferon Regulatory Factor 1
PD-L1: Programmed Cell Death Ligand 1
ISG: Interferon-stimulated Gene
cGAMP: Cyclic Guanosine Monophosphate-Adenosine Monophosphate (cyclic GMP-AMP)
IFIT1: Interferon induced protein with tetratricopeptide repeats 1
RIG1: Retinoic acid-inducible gene I
ENPP1: Ectonucleotide Pyrophosphatase/Phosphodiesterase
TBK1: TANK binding kinase 1
TME: Tumor microenvironment
