# Supplementary material for: Immunomodulatory effects of trastuzumab deruxtecan through the cGAS-STING pathway in gastric cancer cells
Source: Cell Commun Signal. 2024 Oct 24;22:518. doi: 10.1186/s12964-024-01893-3 (PMC11515331; doi:10.1186/s12964-024-01893-3)
Supplement: Supplementary file 1 — Supplementary Material 1. [file 12964_2024_1893_MOESM1_ESM.docx]

**Supplementary Information for**

**Immunomodulatory effects of trastuzumab deruxtecan through the cGAS-STING pathway in gastric cancer cells**

Kyoung-Seok Oh_1_, Ah-Rong Nam_1_, Ju-Hee Bang_1_, Yoojin Jeong_1_, Sea Young Choo_1_, Hyo Jung Kim_1_, Su In Lee_1_, Jae-Min Kim_1_, _2_, Jeesun Yoon_3_, Tae-Yong Kim_1_, _3_, and Do-Youn Oh_1_, _2_, _3_*

_1_Cancer Research Institute, Seoul National University College of Medicine, Seoul 03080, Korea

_2_Integrated Major in Innovative Medical Science, Seoul National University Graduate School, Seoul 03080, Korea

_3_Department of Internal Medicine, Seoul National University Hospital, Seoul 03080, Korea

Do-Youn Oh, MD, Ph.D.

Professor,

Department of Internal Medicine, Seoul National University Hospital, Seoul, Korea

Cancer Research Institute, Seoul National University College of Medicine, Seoul, Korea

101 Daehak-ro, Jongno-gu, Seoul 03080, Korea;

Tel: +82-2-2072-0701; Fax: +82-2-762-9662;

Email: [ohdoyoun@snu.ac.kr](mailto:ohdoyoun@snu.ac.kr)

**
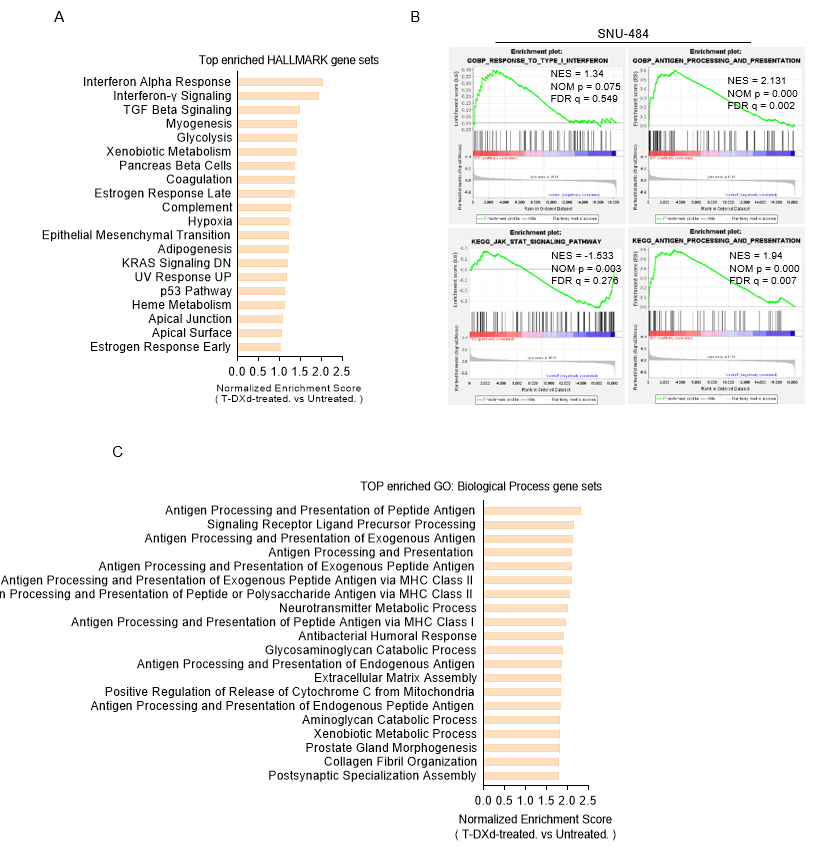
Supplementary Figure 1. RNA-Seq identifies T-DXd treatment-induced gene sets associated with antitumor immunity in HER2-moderate expressing GC cell line, SNU-484.** RNA-Seq analysis was performed using SNU-484 cells treated with vehicle or T-DXd (1 µg/ml) for 72 h. (A) Top 20 enriched Hallmark gene sets ranked by normalized enrichment score from GSEA in response to T-DXd. (B) Representative images from GSEA showing enrichment of gene signatures associated with antitumor immune response. (C) Top 20 enriched GO: Biological Process gene sets ranked by NES from GSEA in response to T-DXd.


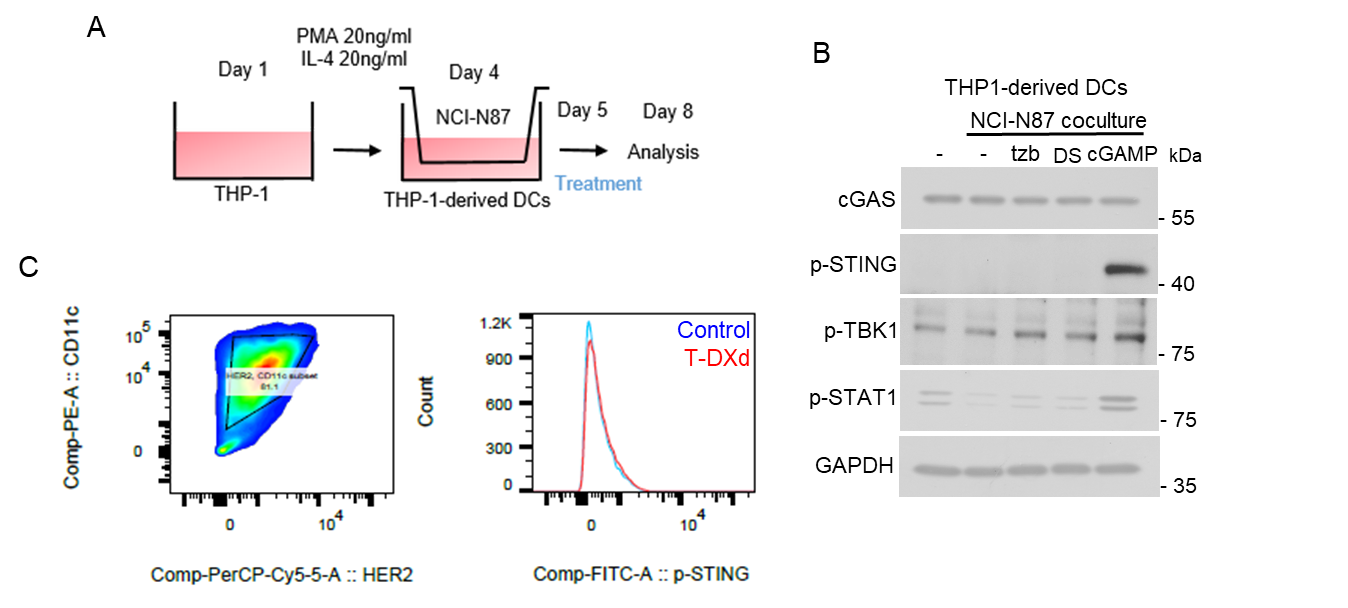


**Supplementary Figure 2. T-DXd fails to induce cGAS-STING pathway activation in TDDCs during indirect coculture in a transwell system.** (A) Schematic diagram illustrating the experimental setup for the indirect coculture of NCI-N87 cells and TDDCs in a transwell system, where direct contact between cancer cells and DCs is prevented. (B) Immunoblot analysis of key components of the cGAS-STING pathway in TDDCs harvested from the lower chamber after 72 h of indirect coculture. (C) Flow cytometry analysis of phosphorylated STING (Ser366) in TDDCs.

**Supplementary Figure 3. T-DXd promotes the release of exosomal dsDNA.** Quantification of exosomal dsDNA isolated from the conditioned medium of NCI-N87 cells treated with 1 µg/ml of trastuzumab or T-DXd for 48 h. Exosomal dsDNA levels were measured using the SpectraMax Quant AccuBlue Pico dsDNA Assay Kit. The sum of two technical replicates is shown as mean ± SEM.

**
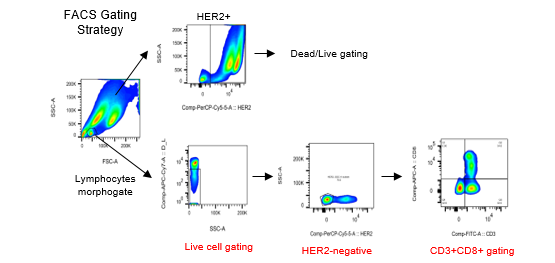
**

**Supplementary Figure 4. Gating strategy for flow cytometry analysis using human PBMCs.** Cancer cells were selected based on size and HER2^+^ expression. Live lymphocytes were selected based on size, Zombie NIR™ Fixable Viability Kit^-^, and HER2^-^ expression. Cytotoxic T cells were selected based on CD3^+^ and CD8^+^ expression.

**
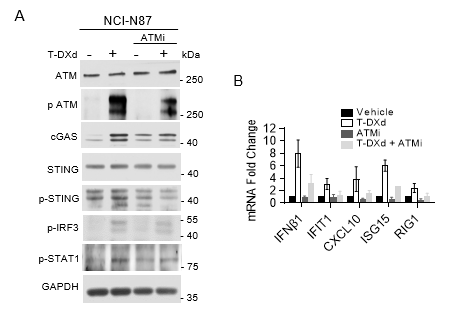
**

**Supplementary Figure 5. ATM inhibition prevents T-DXd-induced cGAS-STING activation in NCI-N87 cells.** (A) Immunoblotting of cGAS-STING pathway in NCI-N87 cells treated with T-DXd (1 µg/ml) with or without AZD0156 (ATMi, 0.1 µM) for 72 h. GAPDH was used as a loading control. (B) RT-qPCR analysis of ISGs in RNA isolated from NCI-N87 cells treated with T-DXd (1 µg/ml) with or without AZD0156 (0.1 µM) for 72 h. Data represent the sum of at least three independent experiments.

**
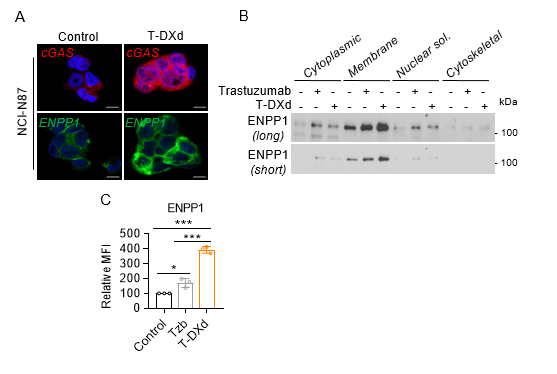
**

**Supplementary Figure 6. T-DXd upregulates ENPP1 expression in HER2-positive GC cells.** (A) Immunofluorescence analysis of cGAS (Red) and ENPP1 (Green) levels in SNU-216 cells treated with T-DXd (1 µg/ml) for 72 h. Representative images from biological duplicates are shown. (B) Immunoblotting of ENPP1 expression in specific cell organelles using lysates isolated from NCI-N87 cells treated with trastuzumab or T-DXd (1 µg/ml). GAPDH was used as a loading control. (C) Flow cytometry analysis of surface ENPP1 expression in NCI-N87 cells treated with trastuzumab or T-DXd (1 µg/ml) for 72 h. Data from biological triplicates are presented as mean ± SEM, *, p < 0.05; ***, p < 0.001.
